# Nonlinearity and distance of ancient routes in the Aztec Empire

**DOI:** 10.1371/journal.pone.0218593

**Published:** 2019-07-17

**Authors:** Igor Lugo, Martha G. Alatriste-Contreras

**Affiliations:** 1 Centro Regional de Investigaciones Multidisciplinarias, Universidad Nacional Autónoma de México, Cuernavaca, Morelos, México; 2 Facultad de Economía, Universidad Nacional Autónoma de México, Ciudad de México, México; Seoul National University College of Medicine, REPUBLIC OF KOREA

## Abstract

This study explores the way in which traveling paths in ancient cultures are characterized by the relationship between nonlinear shapes and path lengths in terms of distances. In particular, we analyze the case of trade routes that connected Aztec settlements around 1521 CE in central Mexico. Based on the complex systems perspective, we used the least cost path approximation to reconstruct a hypothetical large-scale map of routes reproducing physical connections among ancient places. We compared these connections with different spatial configurations and identified the probability distribution functions of path lengths. We evaluated the nonlinearity using the mean absolute error based on the path fitness of simple linear models. We found asymmetrical distributions and positive relationships between those measures. If a path length increases, so does its nonlinearity. Thus, the simple pattern of traveling in the Aztec region is fairly unlikely to be straight and short. Complex pathways can represent most of the ancient routes in central Mexico.

## Introduction

The first land roads are essential to understanding the spatial interactions of ancient civilizations and the dynamics behind the emergence of actual road and urban systems. Organized long distance pathways—trails or footpaths—characterize some of the first nation-states showing higher levels of social, military, and administrative coordination [1–3]. However, in some regions and countries, traces of these paths have disappeared or are unknown. This is the case of ancient routes in the Aztec Empire around 1521 CE in central Mexico. This civilization showed a political integration based on hierarchical settlements—*altepetl*—connected by a transportation system based on human carriers—*tlamemes*—who were organized by long-distance, elite traders—*pochtecah* [4–7]. Archaeological and historical studies have used different scales of analyses searching for empirical evidence that those traders organized the flow of resources—tribute—selecting straight and short pathways [8–11]. These types of paths are associated with foot and canoe travel due to the minimal cost of maintenance and the maximum number of connected locations [12–17]. Even though these path attributes have been used to described ancient pathways, they also represent the ideal connectivity among locations on a flat surface via straight-line routes. Unfortunately, findings have been inconclusive making it difficult to reconstruct a large-scale map of ancient routes that show their best geospatial location and geometric attributes. Therefore, we attempted to recreate an ancient system of routes using the least cost path (LCP) method based on slopes and to compare it with different spatial models in order to determine the relationship between nonlinear shapes and path lengths in terms of distances. These are key properties that describe one state of a set of different and possible routes in the Aztec transportation system.

Owing to the interdisciplinary nature of the analysis, we used the complex systems approach to organize and coordinate data, and used theories and models of economic geography and archaeology to answer the following questions: What is the most likely geospatial location of pre-Hispanic routes in the Aztec region? And what is the relationship between nonlinearity and travel distance of routes in a historical network context? To answer these questions, we applied an intense programming framework based on the Python language and its internal and external libraries for geoprocessing the slope-based LCP, generating spatial graphs, and analyzing data. This data is essential to understanding geospatial interactions of ancient settlements that give rise to complex transportation routes. Then, we used inferential data analysis to identify an universality class in such transport networks. This type of class expresses a large-scale behavior of the system even though their components differ in detail, and it is commonly represented by its statistical distribution [18, 19]. Therefore, based on the resulted distribution, we can infer the common underlying pattern associated with the component dependencies. For example, variables normally distributed describe expected events based on the sum of the system parts, and variables distributed as a power law or exponential delineates extreme and unexpected events based on multiplicative relationships.

Next, we compared the resulting slope-based LCP networks—a set of nodes connected by a set of edges—with hiking, null, and empirical models—reciprocal of Tobler’s cost function [20], straight-line connections, and actual road sections—searching for statistical similarities among probability distribution functions (PDF) of path lengths—the sum of each edge in terms of the distance of the shortest path length. We assumed that these spatial models are proxies for the unknown network of Aztec routes. While the study of White and Barber [21] validated their findings based on extensive archaeological and ethnohistoric data of the region, we used the hiking, null, and empirical models to sufficiently validate our results due to the limited data set. To measure nonlinearity, we computed the mean absolute error (MAE) to evaluate the fitness of simple linear regressions to the shapes of shortest paths, and then we identified their PDFs. We argue that the path length presents a positive relationship to nonlinear shapes: short-distance paths are less nonlinear because of the minimalist assumption of people traveling in local environments, and long-distance paths are curved routes due to complex traveling patterns based on the territory and social interactions. Therefore, this study will enhance our understanding of how the geometric and geospatial attributes of the first pathways could characterize one possible state of initial conditions in the actual spatial organization of urban systems.

This paper has been divided into four sections. The first section explains materials. The second section describes the method based on an interdisciplinary approach. The third section presents the results. Finally, the last section provides discussion of those results and our conclusions.

## Materials

**Fig 1:**
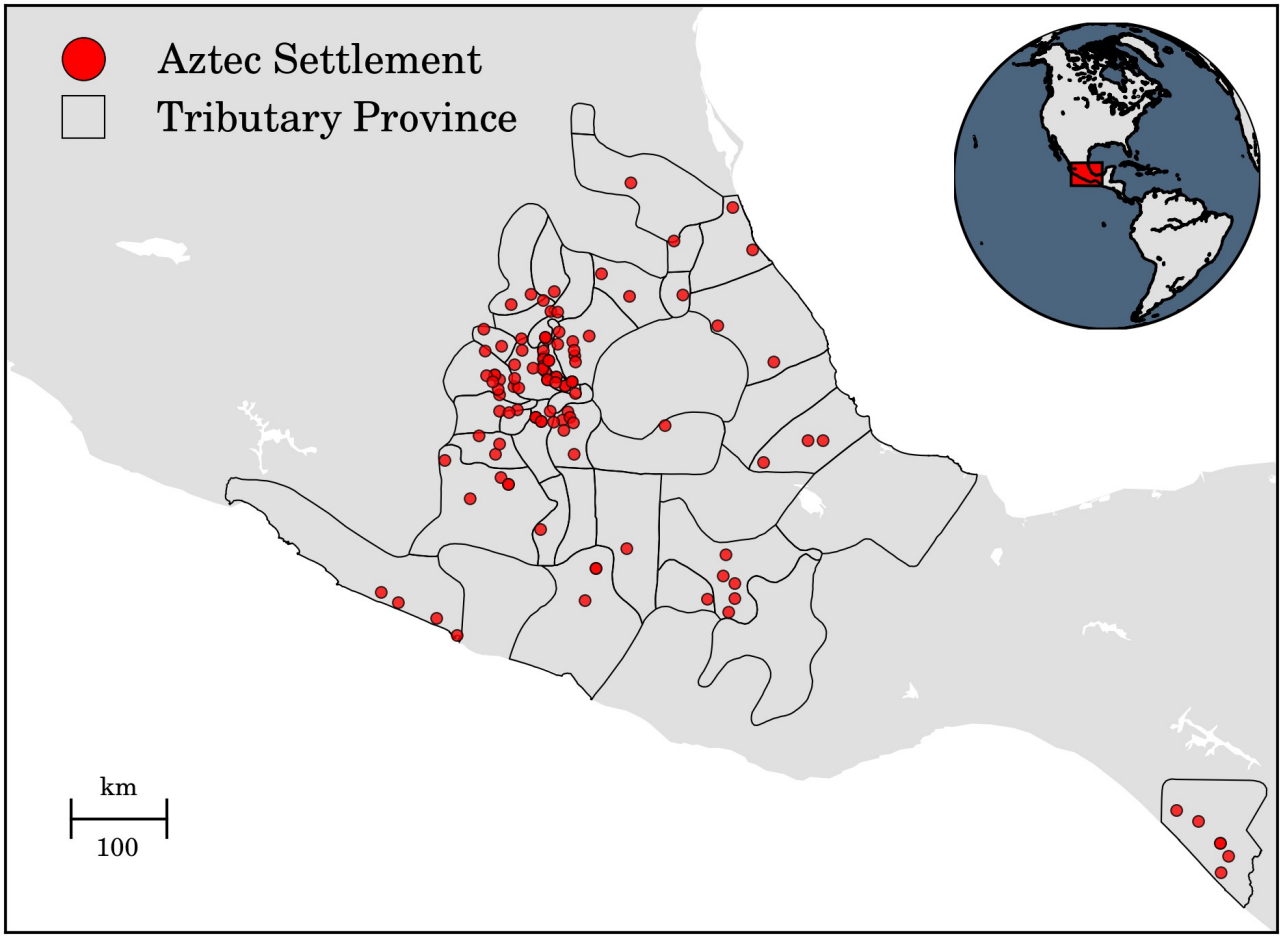
Geography of the region and GIS data of Aztec settlements and tributary provinces. The data is based on Barlow’s study [30] and was provided by the Instituto Nacional de Antropología e Historia (INAH). To show the area of the empire, we use the vector data of tributary provinces.

**Fig 2:**
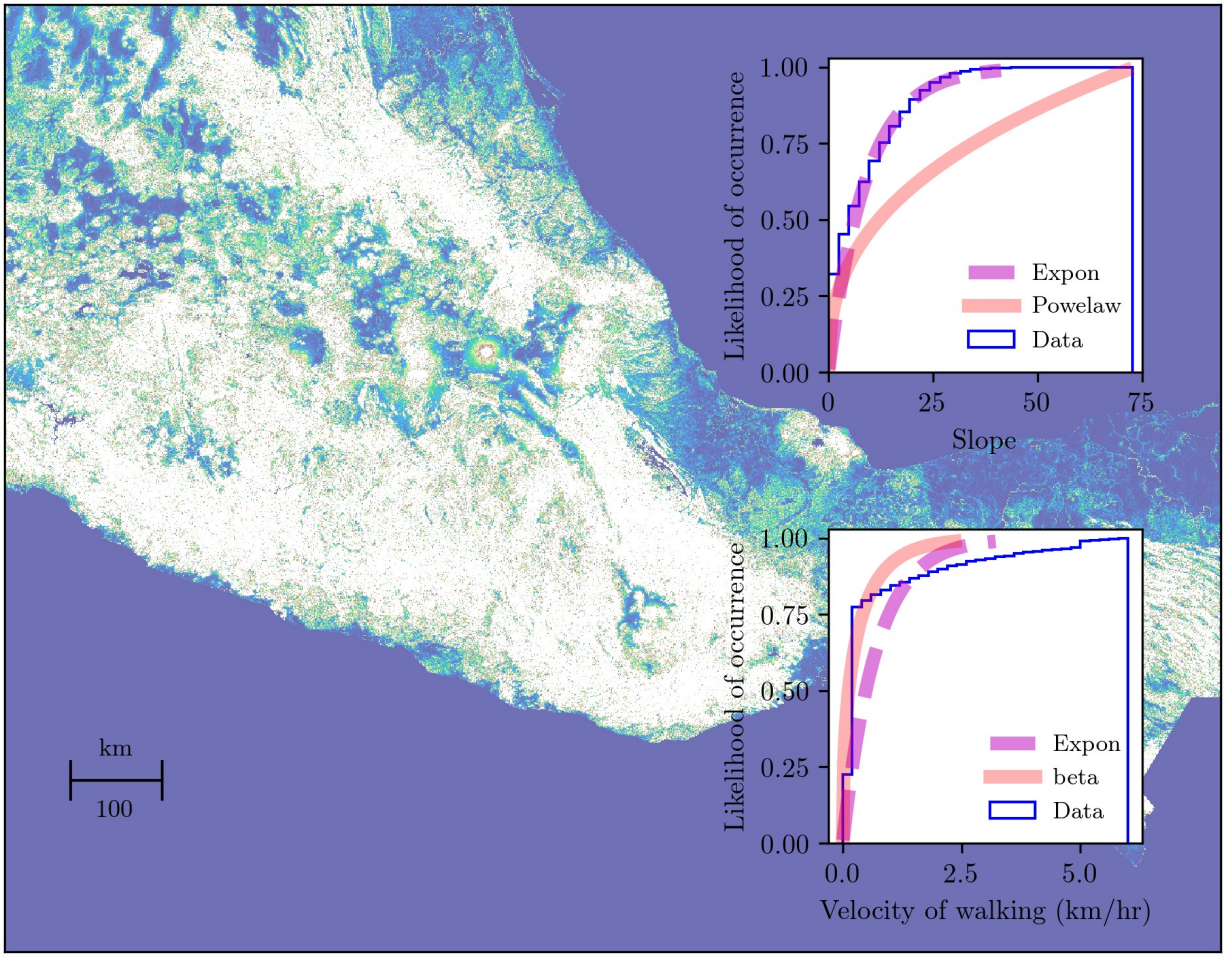
Estimated PDFs of slopes and hiking approximations in the Aztec region. The raster resolution is 222 m. Compared with other continuous distributions, in the slope approach, there are two PDFs that best describe the data: power law and exponential. The former uses a probability density function of the form *f*(*x*, *α*) = *αx*^*α*−1^, for 0 <= *x* <= 1, *α* > 0. The latter shows a probability density function of the form *f*(*x*) = *exp*(−*x*), for *x* >= 0. In addition, the hiking approach—velocity of walking—shows two PDFs that best describe the data: beta and exponential. The beta distribution uses a probability density function of the form $f\left(x,a,b\right)=\frac{\gamma \left(a+b\right)x^{\alpha -1}{\left(1-x\right)}^{b-1}}{\gamma (a)\gamma (b)}$, for 0 < *x* < 1, *a* > 0, *b* > 0. The exponential distribution shows the same probability density function as the slope data. Table 1 shows the estimated parameters, KS test statistics, and first moments of these PDFs. For supporting information, OSFNotebook and S1 Table.

We used two types of materials in this study: geospatial data and Python libraries. The geospatial data is related to the geographic information system (GIS) technology whose computational tools provide the flexibility to organize, analyze, and recreate data of possible ancient land routes [22, 23]. Compared to traditional cartography, which is considered either an artistic representation or a pictorial perception of reality because of its descriptive level and imprecise data, the GIS uses geospatial data to improve the accuracy of measurements and to explore different scales of analysis. Therefore, we used the official vector data of point settlements in the Aztec Empire (Fig 1) and the current geospatial data of road networks [24, 25]. The latter data was used for the comparison of similar network properties because it is associated with the same region and terrain as the slope-based LCP. Additionally, it represents a current version of older roads in the region, i.e., modern road networks emerge from ancient land routes [26–28]. Furthermore, we used the Mexican digital elevation model (DEM) and its slope-dependent approximations (Fig 2) [29].

**Table 1:** Estimations of slope and hiking approximations.

Estimations	Slopes
power law *(best fit)*	
Parameters	(0.3840, -2.7003x10^−25^, 73.0069)
KS test	(0.00072, 0.7749)
First moments	(12.0119, 20.2596, 448.2388, 0.9068, -0.4163)
exponential *(second best)*	
Parameters	(0.0, 8.9561)
KS test	(0.00077, 0.6947)
First moments	(6.2079, 8.9561, 80.2132, 2.0, 6.0)
	**Hiking**
exponential *(best fit)*	
Parameters	(3x10^−323^, 0.6983)
KS test	(0.00038, 0.9592)
First moments	(0.4840, 0.6983, 0.4877, 2.0, 6.0)
beta *(second best)*	
Parameters	(0.4187, 138.9978, -1.0201x10^−27^, 108.0434)
KS test	(0.00039, 0.9557)
First moments	(0.1244, 0.3245, 0.2489, 3.0440, 13.7600)

Estimated parameters: (a, b, loc, scale); KS goodness-of-fit test: (D, p-value); and first moments (median, mean, variance, skewness, kurtosis).

Fig 2 shows transformed slope angles and their hiking calculation. Slopes are associated with two biased distributions: power law and exponential. The graph shows that the exponential distribution best fits the data (S1 Table). Based on the exponential distribution, we can see that there is a 95% chance that the slope data does not exceed an angle of 40°. The hiking data is associated with the exponential and beta distributions. As in the slopes, the exponential distribution best describes the data (S1 Table). Following the studies of Minetti et al., [31] and Llobera and Slukin [32], we assumed that walking or running at an angle of less than 40° is preferred, hence people choose to go around rather than climb uphill in these areas. Consequently, nonlinear shapes characterize the traveling paths over irregular surfaces.

Based on the complex systems approach, we selected a spatial resolution, shown in Fig 2, based on the tradeoff between the information needed to describe a system and its size—the complexity profile [33]. We are interested in describing a large-scale network of ancient routes in the Aztec Empire instead of a single path on a fine scale. In addition, we worked on this resolution because of the tradeoff between the size of the grid cell and the computational tractability of the slope-based LCP algorithm. That is, fine-scale data requires more computational resources. Therefore, by using the slope-based LCP method and the selected resolution, we could sufficiently reproduce a large-scale network of travel by foot. Thus, each of these materials represents the basic data used to simulate pathways among Aztec settlements and to compare them with different spatial models.

The Python libraries provided the computational algorithms and code instructions based on third party packages for geoprocessing using the slope-based LCP algorithm, generating the different spatial networks, and analyzing them. These libraries are Numpy [34], Matplotlib [35], Scipy [36], NetworkX [37], GDAL/OGR [38], Scikit-image [39], and Haversine [40]. These materials are available in the Open Science Framework (OSF) and have been made available to show the reproducibility of our results, project name: Large-Scale Transport Networks in Ancient Civilizations (OSFproject).

## Method

We propose an interdisciplinary approximation to gain insights into the relationship between nonlinearity and distance of ancient paths in Aztec times. We use three types of geospatial approaches to support our method: spatial networks, economic geography and archaeological GIS. About the first two approaches, previous studies have examined the relationship between both in a system of cities [41–43]. For example, a spatial network can represent a system of cities where they are a set of nodes with singular attributes, and edges might represent physical infrastructure or abstract characteristics to connect those cities. In those reports, it was shown how important it is to identify the network configuration based on its statistical attributes before quantifying any type of distance measure and describing spatial interactions.

In addition, the archaeological GIS includes the time and the regional context in the analysis, providing guidance for the appropriate use of data and its interpretation [44, 45]. This approach considers historical information to reconstruct and analyze geospatial systems. In particular, to analyze likely routes in ancient cultures, scholars have used variations of the accumulated cost surface (ACS) algorithm [46–48]. Therefore, the slope-based LCP algorithm has been used as one tool for answering past and present questions about the structure, dynamics, and context of transportation and urban systems.

Our method is a sequence of three processes. The first process recreates possible geospatial configurations of pathways in the Aztec Empire using the slope-dependent cost functions: slopes and their reciprocal of the Tobler hiking approach. They simulate networks of paths that connect ancient settlements. Once these networks are created, the second process generates two spatial networks in which those settlements are connected by different paths. The first network, the straight-line link model, consists of settlements connected by simple line objects. It represents a completed graph because each location is connected to each other. One characteristic of this network is its similarity to random null models due to its PDF of path lengths [43]. The second network, the empirical model, connected Aztec settlements based on the Mexican road network. We associated settlement locations to the closest point in the road network. On completion of this process, we computed the shortest paths and their lengths for every pair of settlements the model.

The last process was to analyze and compare every set of shortest paths in terms of distances by applying inferential data analysis. We identified statistical similarities in the PDFs and measured nonlinear paths computing the MAE. Then, we measured the strength of association between nonlinearity and path lengths by the Spearman correlation coefficient to test the linear assumption of straight and short pathways in the Aztec region.

### Least cost path

One of the most well-known tools in archaeology for studying ancient routes is the LCP. It is defined as the minimum number of route sections that form a path between two locations on a continuous surface [11, 23, 49, 50]. Compared to alternative methods of complex systems—for example, the flow accumulation, the active walker, and the slime mold—the LCP method is a practical way of reproducing geospatial connections among ancient settlements based on basic geospatial data [3, 51–56]. Following the GIS framework, the cost is associated with a grid or array of a region in which each cell contains varying measures related to spatial attributes—in this case slopes and their reciprocal of Tolber’s hiking values, the pace function, given by *t*(*h*) = 0.001/(6 exp(−3.5|*s* + 0.05|)), where *t*(*h*) is the time per hours and *s* is the slope as dh/dx [20, 57, 58]. The goal of the method is to minimize the cost and the distance to connect two locations by a set of adjacent cells. Therefore, this method uses a computer algorithm to solve the problem of human movements in rough terrain.

The computation of the slope-based LCP is associated with a the well-known pathfinding method, in particular, the A* algorithm [59, 60]. Its advantage is the cost function: *f*(*n*) = *g*(*n*) + *h*(*n*); where *n* is the next location on the path, *g*(*n*) is the cost of the path from the initial location to *n*, and *h*(*n*) is the heuristic function that estimates the minimum cost of the path from *n* to the goal. However, like other processes used to extract features from images, this method is highly sensitive to the scale of cells [61]. Therefore, the heuristic function solves the problem of being practical—not optimal or perfect—speeding up the process of finding a solution. We used this algorithm because it is a generic model that sufficiently replicates practical, human movements on complex terrain when traveling from one location to another.

While this slope-based LCP method is based on a complex system approximation, the archaeological perspective uses different LCP specifications. For example, Llobera et al. [62] proposed an analytical framework based on different formulations of the accumulated cost surface, Sherman et al. [63] identified potential transportation routes using a simple specification of the LCP, White and Barber [21] proposed a formulation in which origin and destination points are not required in the analysis to generate a natural-looking travel network, and Güimil-Fariña and Parcero-Oubiña [64] explored the use of the LCP method to identify the main nodes in a network based on existing Roman roads. These specifications, which produce path networks and related information at different scales, use data that restricts their model validation. Therefore, following the suggestion of Verhagen [65] about the integration of GIS in archaeology with other approaches, we consider that the archaeology can complement its analysis using the concept of universality in complex systems—i.e., different system behaviors on a particular scale can be similar even though they differ in details—to select a set of models—theoretical and empirical—to validate their results by comparing similar systems. In addition, these models are important when archaeology and ethnohistoric data are limited.

To begin this procedure, we used the slope angle in degrees and the reciprocal of velocity for each cell of the Mexican DEM model (Fig 2) and the point layer of Aztec settlements (Fig 1). After we loaded the data, we computed the slope-based LCP using the pathfinding algorithm [66]. In particular, we used the *route_through_array* function, selecting two different parameters for generating diagonal and axial moves. These parameters control the size of the length used to weight the path cost—in axial moves, the weighted distance is unity, and in diagonal moves, the weighted distance is $\sqrt{2}$. Consequently, we selected weighted distances to minimize the path cost. Then, we generate two spatial network based on slopes, and two models based on the hiking values by iterating over every location of the Aztec settlements. Once the data was obtained, we saved it as linestrings in a shapefile (see the supporting information, OSFNotebook).

### Spatial models

Null models provide the data and information for comparing one specific network to different situations by identifying similar or dissimilar structural features [43, 67]. Consequently, we used two types of null spatial models to compare the slope-based LCP results.

The first model was a straight-line network where a single line connects each settlement to every other settlement. The second model was an empirical network based on the Mexican road system. Both configurations represent good comparatives because they represent theoretical and empirical results. For example, the straight-line network symbolizes the ideal connectivity in which the distance between locations is the minimum as in a flat surface. On the other hand, the empirical network shows modern road sections used by land transportation.

The process of loading geospatial data and generating networks used the GDAL, NetworkX, and Haversine libraries. The data of Aztec settlements was used to form the straight-line network. It shows direct connections among locations; its edges are associated with a distance attribute computed by the Haversine [40] library—the distance between two points on Earth. Next, the process of generating the empirical network used the data of Aztec settlements and the road network. The goal was to identify and join closer point settlements to linestring points. We converted road data to a network where each linestring was divided into single lines—a pair of nodes connected by an edge. Because this process used a large amount of memory when computing, we decided to round the geospatial coordinates (latitude/longitude) to three digits. The output was a network that forms the largest connected component, which is a close representation of the original data. Finally, closer points were identified and joined to nodes in the network (see the supporting information, OSFNotebook). Therefore, both configurations showed different properties for comparing the slope-based LCP results.

### Data analysis

We used an inferential data analysis method to identify similarities between the simulated patterns in the slope-based LCP networks and the datasets based on spatial models. Path lengths and MAEs were computed, hence their PDFs were identified. After this analysis, we determined their statistical association.

The first step in this process was to compute the shortest paths and their lengths in terms of distance per network. We used Dijkstra’s algorithm in which the source and target were nodes associated with the Aztec settlements, and weights were related to the Haversine distance [68]. The output was a dictionary object in which the key was a tuple containing the source and target points and the values were a tuple of the path—a list of geospatial nodes—and its length (see the supporting information, OSFNotebook). After this step, we identified the PDF of lengths in each network based on the Kolmogorov-Smirnov (KS) goodness-of-fit test computed by maximizing a log-likelihood function [69, 70]. We used a collection of continuous distributions to generate observed random variables and compared them to a given distribution. Such distributions were: beta, exponential, exponential Weibull, gamma, Gilbrat, log-normal, normal, Pareto, power law, Weibull max, and Weibull min. Therefore, this statistical test provides rounded, detailed illustrations of the type of bias distributions across scales—for example, it reveals the association between extreme values of path lengths and the topography [43].

Next, we identified nonlinear paths with the MAE by fitting a simple linear regression to geospatial paths in which the variables are their coordinates (*x*, *y*). This measure shows the average vertical distance between each point and the estimated line, $\frac{\sum_{i=1}^{n}\left|y_{i}-x_{i}\right|}{n}$. If the MAE values are closer to zero, pathways are best represented by straight-lines, otherwise, pathways are far from linear. Once MAEs have been computed, their PDFs were identified by using the KS goodness-of-fit test (see the supporting information, OSFNotebook).

Finally, measures of the path length and the MAE in terms of distances were analyzed by the Spearman correlation [71]. We looked for a positive correlation implying that as path length increases, so does nonlinearity.

## Results

**Fig 3:**
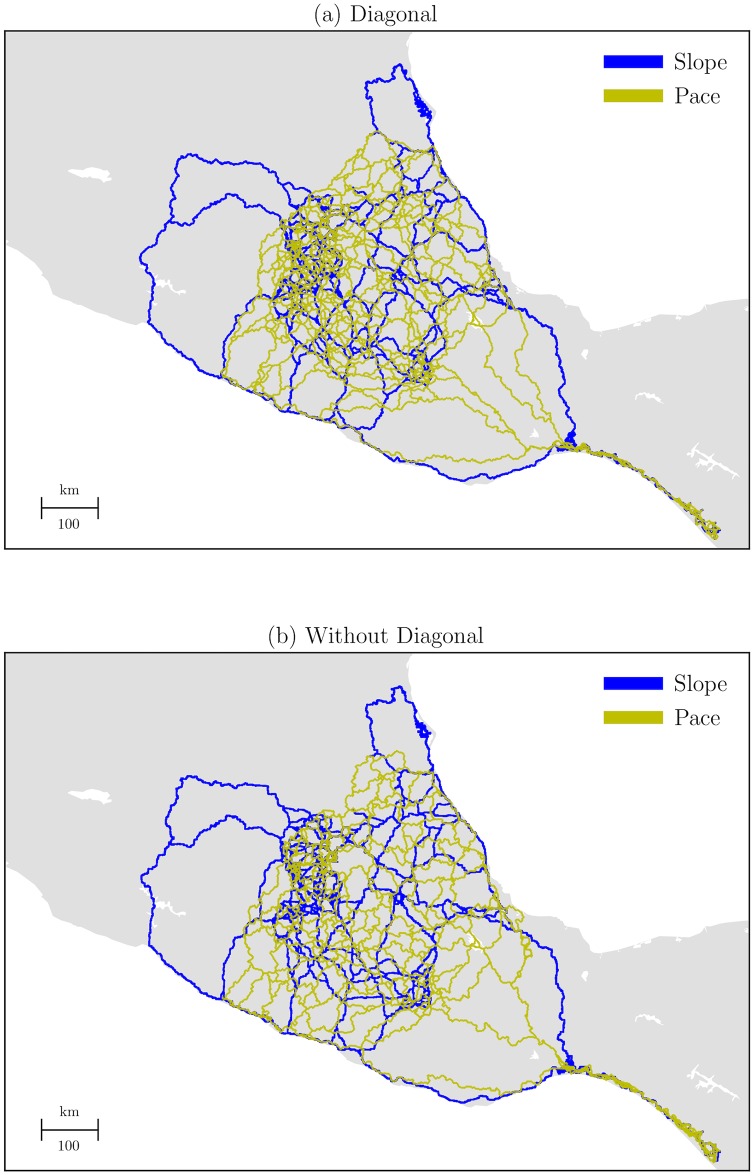
Geospatial location of the slope-based LCPs in the Aztec Empire. Subfigures show 111 point settlements. Subfigure (a), slope data shows 15, 112 nodes and 15, 769 edges; and the reciprocal of Tobler’s data shows 19, 945 nodes and 25, 082 edges. Subfigure (b), slope data shows 20, 454 nodes, and 21, 343 edges; and the reciprocal of Tobler’s data shows 20, 027 nodes and 27, 524 edges.

To understand the probable large-scale network of pathways in which human carriers transported trade and tribute across the Aztec Empire, we simulated trade routes by the slope-based LCP method. Fig 3 shows the resulting spatial networks that connect ancient settlements.

From the figure above, we can see that the geospatial locations of pre-Hispanic routes show two patterns. The first, based on slopes, presents well-defined routes in which short-distance paths show different shapes and locations, and while long-distance paths show similar shapes and locations. According to Hassing [8], there were different types of routes due to their scale: *ohtli*—roads inside or close to main settlements; *ohpitzactli*—trails linking close locations, subdivided by *ixtlapalohtli* (shortcuts) and *ichtacaohtli* (secret); and *icxiohtli*—footpaths for large-scale trajectories. The second, based on the reciprocal of speed data, shows straight-type paths that are widely dispersed in the territory. This suggests that short-distance and long-distance paths were less nonlinear because of the velocity of walking. Therefore, the data suggests that the terrain type and the proximity of settlements affect the shape and location of paths.

**Fig 4:**
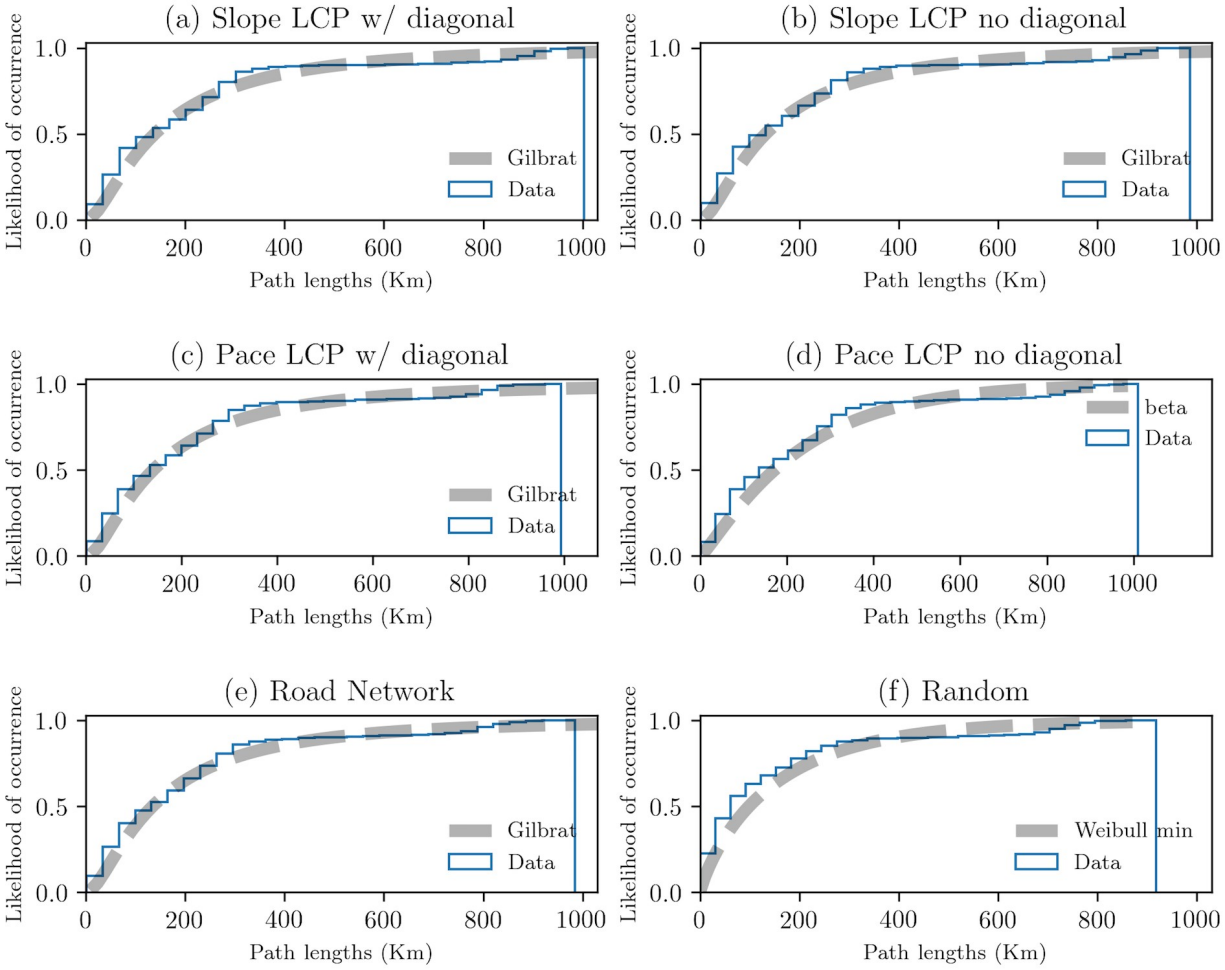
CDFs and best fit of path lengths. Data in subfigures (a), (b), (c) and (e) are best described by a Gilbrat distribution function with a probability density function of $f\left(x\right)=\frac{1}{x\sqrt{2\pi }}exp\left(-\frac{1}{2}\left(log{\left(x\right)}^{2}\right)\right)$. Data in subfigure (d) is best described by a beta function with a probability density function of $f\left(x,a,b\right)=\frac{\gamma \left(a+b\right)x^{\alpha -1}{\left(1-x\right)}^{b-1}}{\gamma (a)\gamma (b)}$, for 0 < *x* < 1, *a* > 0, *b* > 0. Data in subfigure (f) is best described by a Weibull min distribution function with a probability density function of *f*(*x*, *c*) = *cx*^*c*−1^*exp*(−*x*^*c*^), for *x* > 0, *c* > 0. Table 2 shows the first moments of the data. For supporting information, see S2 and S3 Tables.

To clarify and identify differences in paths, we compared the above results with the spatial models—networks of straight-lines and road connections. We analyzed path lengths and identified statistical similarities based on their cumulative distribution functions (CDFs) (Fig 4).

**Table 2:** First moments of Fig 4.

CDF	Subfigures	First moments
Gilbrat	(a)	(138.9784, 231.7560, 95534.1864, 6.1848, 110.9363)
	(b)	(132.4205, 220.8753, 86839.0007, 6.1848, 110.9363)
	(c)	(140.8446, 233.8770, 96059.6923, 6.1848, 110.9363)
	(e)	(134.3634, 224.1903, 89554.0420, 6.1848, 110.9363)
beta	(d)	(171.6162, 233.0946, 45721.1098, 1.8460, 5.1119)
Weibull min	(f)	(98.8861, 161.5328, 34636.4900, 2.4672, 9.5397)

First moments: (median, mean, variance, skewness, kurtosis).

Fig 4 shows the CDFs best described by skewed distributions in which larger values suggest complex path shapes (S2 and S3 Tables). In the slope data, there is an 85% chance that path lengths do not exceed 400 km in the spatial networks. This indicates that larger values of path length are generally presented. In the pace data, there is an 90% chance that path lengths do not exceed 400 km, indicating the same as the slope data. Interestingly, there were similar CDFs among the data, even though they have different spatial configurations. In particular, subfigure (e), which is based on empirical data, shows a shape similar to that of other data simulated with spatial models. These results suggest that the terrain is the common feature that has affected human mobility in the region.

**Fig 5:**
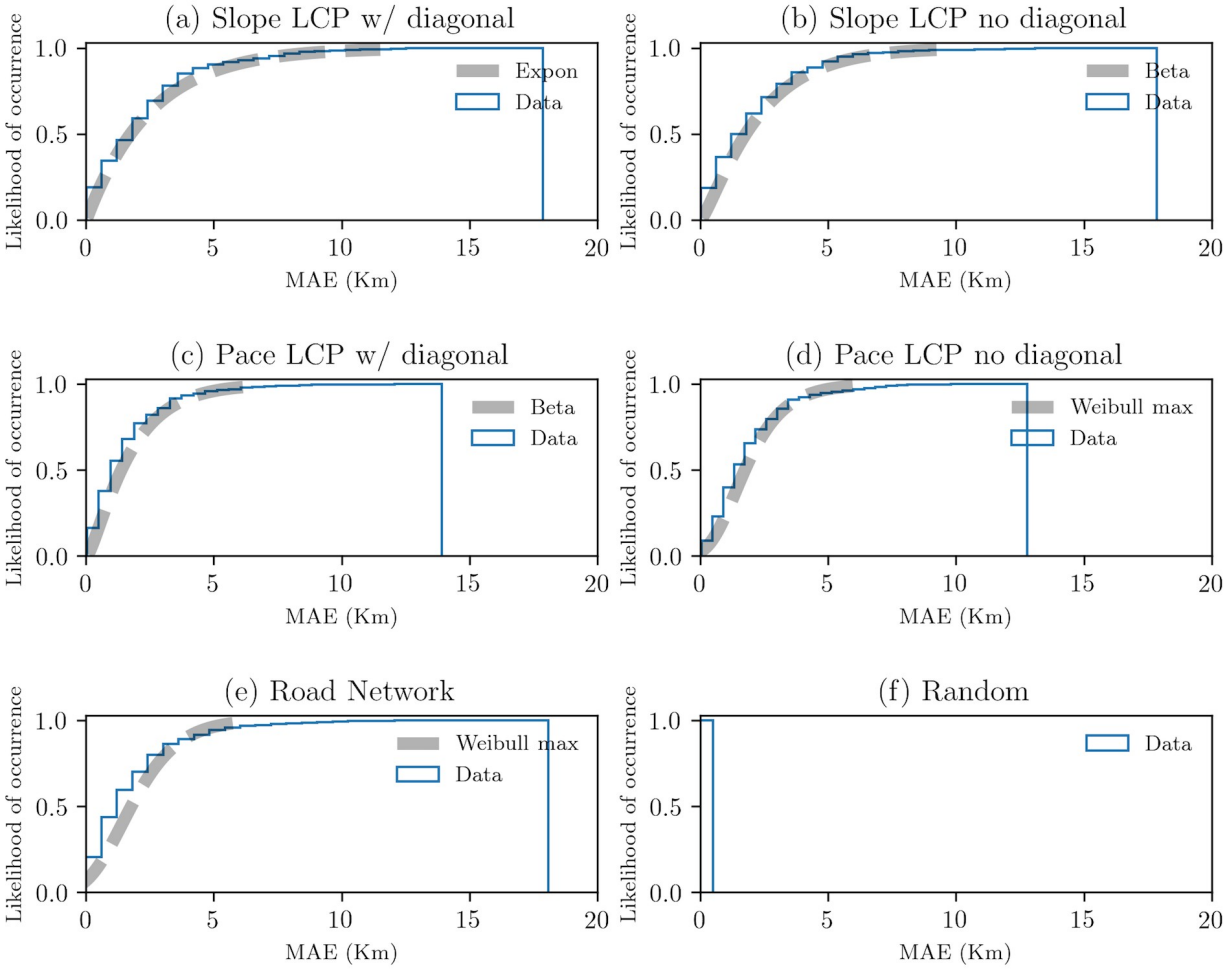
CDFs and best fits of MAE data. Data in (a) is best described by an exponential distribution function with a probability density function of *f*(*x*) = exp(−*x*), for *x* >= 0. Data in (b) and (c) is best described by a beta distribution function with a probability density function of $f\left(x,a,b\right)=\frac{\gamma \left(a+b\right)x^{\alpha -1}{\left(1-x\right)}^{b-1}}{\gamma (a)\gamma (b)}$, for 0 < *x* < 1, *a* > 0, *b* > 0. Data in (d) and (e) is best described by a Weibull max distribution function with a probability density function of *f*(*x*, *c*) = *c*(−*x*)^*c*−1^*exp*(−(−*x*)^*c*^), for *x* < 0, *c* > 0. Table 3 shows the first moments of subfigures. For supporting information, see S4 and S5 Tables.

Next, the nonlinear path shapes were computed with the MAE and their CDFs were identified (Fig 5). The data are best described by skewed distributions, excluding subfigure (f), in which larger MAE values are associated with worse linear fits (S4 and S5 Tables).

**Table 3:** First moments of Fig 5.

CDF	Subfigures	First moments
exponential	(a)	(1.7560, 2.5209, 6.2143, 2.0, 6.0)
beta	(b)	(1.7937, 2.3546, 4.1545, 1.7524, 4.6067)
	(c)	(1.3861, 1.7824, 2.1848, 1.7003, 4.3368)
Weibull max	(d)	(1.7520, 1.9692, 1.7477, -7.6640, -3733415.5207)
	(e)	(1.7770, 1.9620, 2.2202, 0.7454, 0.8681)

First moments: (median, mean, variance, skewness, kurtosis).

As shown in Fig 5, slope data shows an 80% chance that the MAE values do not exceed 4 km, suggesting that a large number of paths differ from linearity to non-linearity over short distances. Pace data shows an 90% chance that the MAE values do not exceed 5 km. This suggests that the non-linearity is a common attribute over short distances. Therefore, geospatial paths with nonlinear shapes permeate the network configurations.

**Fig 6:**
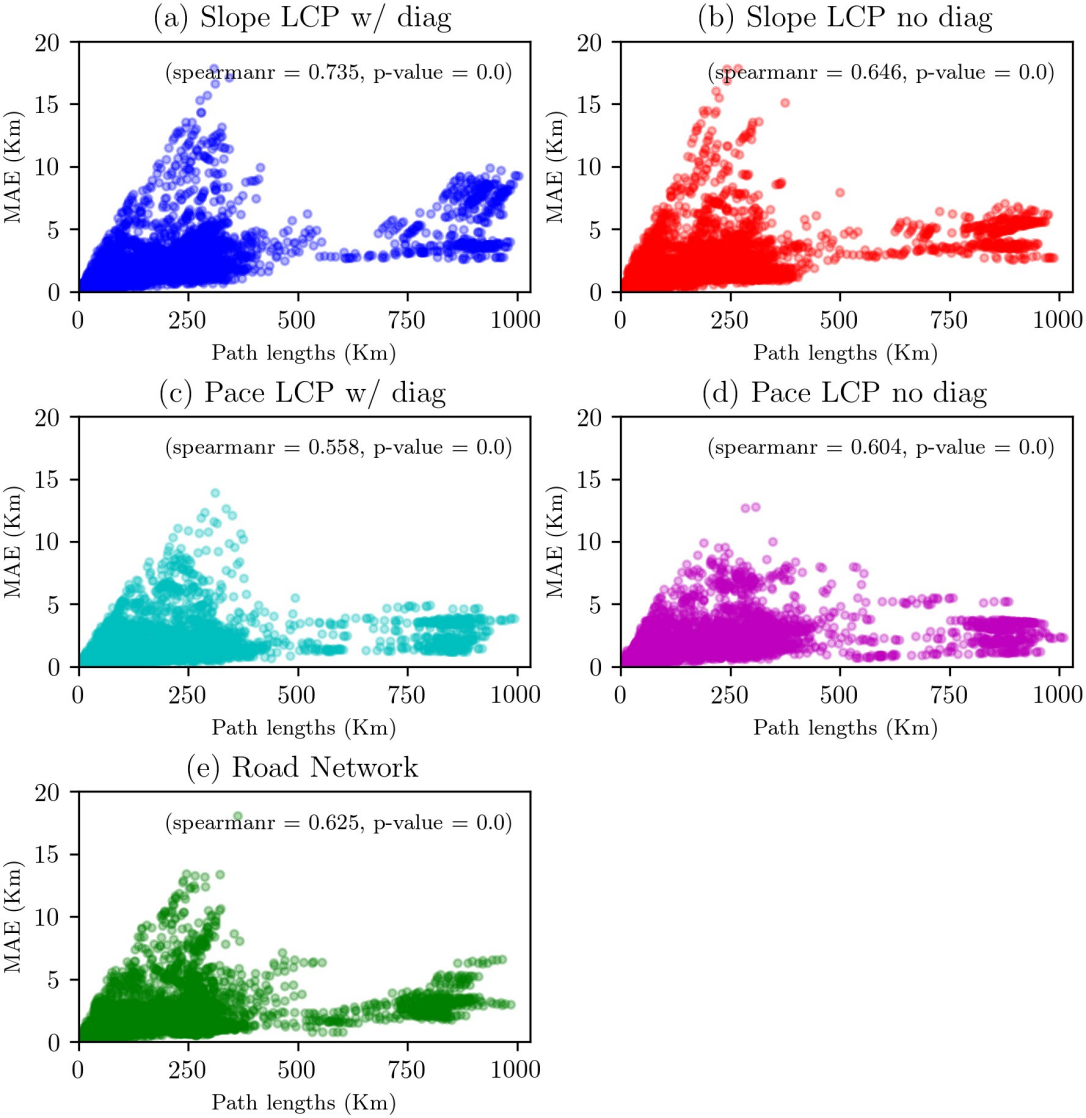
Scatter plot and Spearman rank-order correlation of path lengths and MAE of shortest paths per network configuration.

Finally, to understand the relationship between the path length and shape of paths, we used a correlation analysis (Fig 6).

Fig 6 compares the correlation analyses of different networks. All the cases show positive correlations between the path length and MAE, implying that as path length increases, so does its nonlinearity. In the case of slopes, there are two interesting aspects before and after a path length of 400 km. First, the trend before such a length is more widespread than that of higher values. For example, between 0 and 5 km of the MAE, we can see a higher concentration of data, suggesting that shortest paths are far from linear at about a maximum of 5 km, and higher MAE values of 5 km show an increasing trend with a maximum value around 19 km. Second, path length values higher than 400 km show a slight trend of concentrated points, suggesting that the relationship between path length and MAE shows a tight fluctuation. In the case of pace data, the scatter shows clear and positive trends. Values of path lengths less than 500 km and from 0 to 10 Km of the MAE show higher concentration of data. It suggests that a set of long-distance paths are less nonlinear. Consequently, the subfigures show a Spearman correlation coefficient with close values, even though they represent different geospatial networks. These results confirm a significant relationship between the length and shape of paths, thereby rejecting the linearity assumption of straight and short paths in the region.

## Discussion

The linearity assumption, applied to understand ancient routes, suggests that a straight-line is the easiest and fastest way to connect two locations on different surfaces. However, our results showed that traveling across irregular surfaces is complex and far from linear. In particular, the slope-based and the pace LCP methods reconstructed large-scale networks characterized by biased distribution functions of path lengths and MAEs. They showed a positive statistical association, suggesting that path lengths and nonlinear shapes are correlated. Similar results were found in the empirical model. Surprisingly, the random model showed a closer CDF shape of path lengths than the others, but it was identified by a different probability function. This difference suggests that this configuration partially describes the relationship between those measures. Therefore, we sufficiently described one state of a transportation system in the region that could represent an earlier route network. The path lengths and their nonlinearity are key properties to understand this network due to the assumption of human movements across such terrain. There is a positive relationship between those keys that represent the norm for traveling in complex territories. Moreover, the statistical distributions of path lengths show the presence of skewed distributions similar to that of a universality class—i.e., a power law. The Gilbrat distribution, which is a special case of the log-normal distribution, suggested dependencies between path lengths and locations that give rise to this type of spatial networks.

Based on this information, we can infer that one possible state of ancient routes in the Aztec civilization was nonlinear, which is still the case today in the Mexican road network. Because of the transportation system, the complex landscape, and social interactions with other tribes—Tarascans, Yopes, and Tlaxcaltecas—the best choice to travel around the empire was through nonlinear pathways. These paths were likely preferred not only in mountainous areas, but also in the plains because of the low cost of maintenance. This cost is associated with paths without the construction of hard surfaces, even though there was evidence of paved routes in particular locations—for example, Tenochtitlan. Then, human carriers selected the route based on their local knowledge and not a well-established path. Therefore, these types of ancient routes operated satisfactorily for a long time, promoting the expansion and control of the empire. Nevertheless, they were not exempted from important failures in the transportation system which could compromise the network—for example, the Spanish attack on Tenochtitlan suggested an important cut-off in the flow of resources that led to the collapse of the Aztec Empire.

Considerable attention must be paid when describing coastline paths. Traveling near the coastline suggests that linear paths were preferred because of the use of canoes. This type of transportation system implies less effort for the human carriers and more economic benefits to the empire because the trade was faster and of higher volume—for example, trading with Central and South American civilizations. One of the issues that emerges from these findings is the presence of natural harbors in the network of Aztec routes. However, future research should be undertaken to investigate ancient maritime routes and their interplay with land transportation networks.

## Conclusion

Our findings suggest that the easiest way of traveling in the Aztec Empire was via nonlinear pathways. Their path length distribution follows a universal class of complex system behaviors frequently characterized by the Gilbrat distribution. These nonlinear shapes, positively correlated to their path lengths, typify ancient routes. Such a correlation provides some support for the conceptual premise that transportation systems depend on the geography and social interactions to define the best routes for traveling. That is, if a route is associated with small values of the average absolute difference of distances between modeled and straight paths, its path is likely to be linear because of local knowledge and social coordination between close settlements; however, if a route is associated with higher values of such an absolute difference, its path is likely to be nonlinear due to the geographic conditions and lack of social coordination. Then, from ancient times till today, a large number of transportation routes have followed nonlinear paths. This is a fundamental spatial attribute that has shaped the configuration of actual urban systems.

## Supporting information

S1 Table Best fit and KS test of slopes. (EPS)

S2 Table Best fit and KS test of path lengths. (EPS)

S3 Table Best fit and KS test of path lengths (continued). (EPS)

S4 Table Best fit and KS test of MAE. (EPS)

S5 Table Best fit and KS test of MAE (continued). (EPS)
